# Therapeutic Potential of Direct Clearance of the Amyloid-β in Alzheimer’s Disease

**DOI:** 10.3390/brainsci10020093

**Published:** 2020-02-10

**Authors:** Dong Eun Kim, Ronny Priefer

**Affiliations:** Massachusetts College of Pharmacy and Health Science University, Boston, MA 02115, USA; dkim4@stu.mcphs.edu

**Keywords:** Alzheimer’s disease, amyloid-β, lipoprotein receptor-related protein 1, receptor for advanced glycation endproducts, cerebrospinal fluid

## Abstract

Alzheimer’s disease (AD) is characterized by deposition and accumulation of amyloid-β (Aβ) and its corresponding plaques within the brain. Although much debate exists whether these plaques are the cause or the effect of AD, the accumulation of Aβ is linked with the imbalance between the production and clearance of Aβ. The receptor for advanced glycation endproducts (RAGE) facilitates entry of free Aβ from the peripheral stream. Conversely, lipoprotein receptor-related protein 1 (LRP1), located in the abluminal side at the blood–brain barrier mediates the efflux of Aβ. Research on altering the rates of clearance of Aβ by targeting these two pathways has been extensively study. Additionally, a cerebrospinal fluid (CSF) circulation assistant device has also been evaluated as an approach to increase solute concentration in the CSF via mechanical drainage, to allow for removal of Aβ from the brain. Herein, we provide a brief review of these approaches that are designed to re-establish a homeostatic Aβ balance in the brain.

## 1. Introduction

Alzheimer’s disease (AD) is a neurodegenerative disease characterized by progressive memory loss and defective cognitive abilities. AD is the sixth leading cause of death for patients 65 years and older within the US [1]. Due to no definitive cause for AD, there remains a misconception that AD is only an age-related disease. In the US, there are approximately 200,000 AD patients under 65 years of age [1]. There are two primary hypotheses regarding the development of AD: (1) the amyloid hypothesis, which is the idea that the accumulation of Aβ plaques is the primary cause of AD; and (2) the tau hypothesis, which is the idea that the principle causative substance of AD is the hyperphosphorylated Tau protein, leading to neurofibrillary tangles (NFTs). Amyloid plaques are generated by extracellular aggregation of insoluble amyloid-β (Aβ) peptides in the brain, whereas NFTs are bundles of filamentous protein, usually in the cytoplasm of neurons [2]. Increased levels of neurotoxic Aβ peptides and NFTs in the AD brains have been shown to contribute to the progression of the disease. Due to its unknown pathophysiology, there are currently no curative therapies for AD. All approved drugs are designed only for symptom improvements, such as minimizing memory loss and cognitive improvement. Moreover, drug approval of AD has the highest failure rates, of approximately 99% from 2004 to 2009 [3]. Researchers have put significant efforts toward investigating new approaches of specific targeting of the amyloid plaques. Aβ peptides, composed of 36 to 43 amino acids, are derived by the cleavage of the amyloid precursor protein (APP) expressed in brain endothelial cells, astrocytes, and neurons [4,5]. After cleavage by β-secretase, followed by γ-secretase, the Aβ peptide can aggregate together to form plaques [4,5]. The imbalance between Aβ production and clearance can cause the Aβ plaque production potentially contributing to the devastating symptoms of AD. Increasing the clearance of the Aβ peptides in the brain could directly reduce the progression of the disease and even possibly prevent AD. The blood–brain barrier (BBB) not only plays an essential role in the protection of toxic molecules from entering, but it also can trap big neurotoxic molecules in the brain. Lipoprotein receptor-related protein 1 (LRP1), located at the abluminal side of BBB [6], is a multifunctional scavenger receptor that regulates the removal of Aβ peptides from the brain’s interstitial fluid (ISF) and transports them through BBB into the systemic blood. LRP1 mediates gene expression by coupling with other proteins or receptors [6], regulates ligand internalization, and activates signal transduction pathways [5]. Once free Aβ in the circulation binds to soluble LRP1 (sLRP1) [6], this complex can be hepatically eliminated from the body [5]. In AD patients, the downregulation of LRP1 reduces the amount of the Aβ efflux, thus causing an accumulation of Aβ. The direct agonistic targeting of LRP1 could increase the clearance of the Aβ from the brain, which can later be eliminated by the liver. Free Aβ can also reenter the ISF of the brain from peripheral circulation via the receptor for advanced glycation endproducts (RAGE), located at the BBB. RAGE bound by advanced glycation endproducts (AGE) products mediates gene expression, to increase the level of proinflammatory cytokines and microglia activity [7]. These activities lead to apoptosis of neurons and the accumulation of Aβ. It is hypothesized that directly blocking RAGE could decrease Aβ accumulation, which could prevent the formation of plaques in the brain. Thus, RAGE antagonist may be an approach to clearing out the Aβ for minimizing memory loss and cognitive disability in the pre-phase of AD patients.

## 2. Receptor for Advanced Glycation Endproducts

The levels of Aβ have been shown to increase and accumulate in AD patients and aging brains, and this may be due to the new Aβ influx crossing the BBB via RAGE. Aβ binds to RAGE, causing oxidative stress that leads to mitochondrial dysfunction, energy dysfunction of neuronal cell [6], and an alternation of signal mechanism, including a mitogen-activated protein (MAP) kinase pathway [8]. RAGE is a member of the immunoglobin supergene family expressed on various cell types, mostly in the brain [9]. It is activated by AGEs, S100 calcium-binding protein B (S100B), and high mobility group box 1 (HMGB1), causing the accumulation of Aβ [6,10]. RAGE also magnifies the inflammatory response inside of the brain by releasing cytokines, to cause neuronal damage and apoptosis [11].

**Figure 1:**
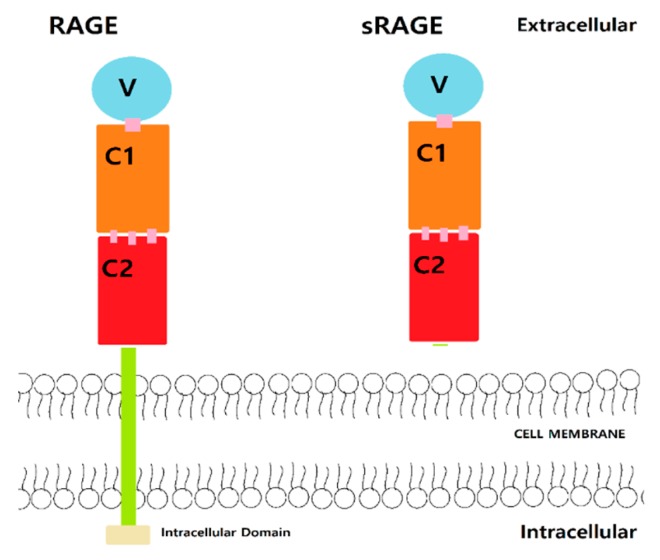
Structure of receptor for advanced glycation endproducts (RAGE) and soluble receptor for advanced glycation endproducts (sRAGE).

RAGE is composed of intracellular, extracellular, and transmembrane domains. The extracellular domain interacts with the ligand and has a V-, C1-, and C2-type domain (Figure 1). The inter-domain hydrogen bonds and hydrophobic interactions [6,12] connect the V and C1 domains, which are the binding sites of AGE, S100 protein, and Aβ [6,12]. The C2 domain acts as a bridge to connect between the C1 domain and the transmembrane region. The V domain is mostly composed of Arg and Lys residues and thus highly positively charged [6,12]. RAGE is considered as a pattern-recognizing receptor, interacting with highly negatively charged binding sites of AGEs, S100 protein, and Aβ [13]. Aβ residues 17–23 display electrostatic interactions with the V domain of RAGE [14]. The hydrophobic region of V and C1 domain of RAGE contains Ile, Ala, Pro, Leu, Val, Trp, and Tyr and interacts with the hydrophobic area of Aβ [6,12]. Potential compounds that could interact with RAGE at the binding sites may hinder Aβ interactions and thus prevent transportation into the brain.

AGEs formation actively accelerates the conversion of Aβ peptide monomers to oligomers [15]. Transition metal, such as copper and iron, cause AGEs to accelerate the oxidation of glucose and fructose and aggregate the Aβ peptide, to form higher molecular-weight Aβ peptides [15,16]. AGE and Aβ activate RAGE, to increase the expression of proinflammatory cytokines tumor necrosis factor alpha (TNF-α), interleukin 6 (IL-6), and macrophage colony-stimulating factor (M-CSF) [17]. Subsequently, RAGE triggers an intracellular signaling pathway through activating transcription factor κB (NF-κB)-dependent pathways [15,18]. Intracellular increases of methylglyoxal can also inhibit mitochondrial respiration, disturbing glucose metabolism, and can lead to diabetes [15]. AGE also enhances its effect and its half-life by causing defective Aβ clearance [15]. The microglia from transgenic presenilin-1 (PS1)-APP mice yield an inverse correlation between cytokine production and Aβ clearance [15]. 

Microglia and astrocyte cultures taken from rapid brain autopsies of AD patients had an increase in cytokines and inflammatory peptides during exposure to Aβ1–42 [7,17]. RAGE-Aβ complexation displays an increase in proinflammatory upregulating and activates protein inflammatory genes, such as indoleamine-2,3-dioxygenase and kynureninase involved in the formation of neurotoxic quinolinic acid, S100A8, and inflammatory chemokine [7]. The proinflammatory mediators regulate the degree of Aβ deposit and microglia activity [19]. The exact role of microglia in clearing Aβ is still not fully understood; however, microglia seem to be involved in the proinflammatory pathway via NF-κB activation and are the main source of cytokines such as TNF-α and IL-β [8,20]. Mutant amyloid precursor protein (mAPP) transgenic mice induce the protein inflammatory cytokine by microglia and astrocytes and amplify the Aβ deposit [21]. In APPsw^+/−^ mice and AD patients, reduction in RAGE activation on microglia decreases the release and secretion of cytokines [6,22,23]. The decline in neuroinflammation prevents apoptosis of neurons, improves neuronal function, and leads to memory and learning improvement [6,23]. RAGE antagonists could theoretically be effective by regulating both Aβ deposits and microglia activity, to reduce the level of proinflammatory cytokines.

The introduction of mutant RAGE has been demonstrated to work as a decoy receptor by decreasing Aβ binding to RAGE. The mutant RAGE could decrease inflammation and increase cerebral blood flow. Notably, soluble RAGE (sRAGE) is one of the mutant forms from splice variants of the gene AGER (Figure 1) [6,11,24] and can antagonize the effects of RAGE ligands by decoying and decreasing the inflammation level and increasing cerebral blood flow. This may reduce neuroinflammation and the level of Aβ to prevent further complications or progression of AD [11,14]. RAGE can also interact with itself through the C1 domain by self-oligomerization. Furthermore, sRAGE can form hetero-oligomerizes with RAGE, thus preventing binding of Aβ to RAGE, and causing reducing effects of Aβ [6,12]. Moreover, sRAGE could be a therapeutic target to mediate the effect of Aβ-RAGE as a decoying agent.

Researchers have been developing RAGE antagonists as a therapeutic target of treating mild AD. The RAGE-blocker Azeliragon (Table 1) demonstrated the safety and effectiveness for 18 months in phase II clinical trial [23,24,25,26,27,28]. However, in 2018, during the phase III clinical trial, Azeliragon failed to prove effectiveness in mild AD patients compared with the placebo group [25,26,27,29,30]. There were no significant differences in treatment over placebo group in co-primary endpoints of Alzheimer’s Disease Assessment Scale cognitive subscale (ADAS-cog) and clinical dementia rating scale sum of boxes (CDR-sb) after 18 months of treatment [25,27]. Further analysis of STEADFAST revealed declines in cognitive impairment, dementia, and inflammation in a patient with mild-to-moderate AD and diabetes patients [25].

## 3. LDLR-Related Protein 1

Among the LDL receptor (LDLR) family sharing a structural and functional similarity, the role of the LDLR-related protein 1 (LRP1) is mostly related to accumulation and clearance of Aβ [31]. LRP1 serves as a scavenger receptor which enables transportation and metabolism of ligands such as apolipoprotein E (apoE), apolipoprotein J (apoJ), and α2-macroglobulin (α2M) [32]. These ligands possibly bind to the Aβ peptides, and then LRP1 mediates the clearance of Aβ at the abluminal side of the BBB [32]. For instance, the apo J can enhance clearance of Aβ through LRP1 at the BBB, whereas apo E interrupts Aβ clearance at the BBB by sending apo E- Aβ complex from LRP1 to very-low-density lipoprotein receptors (VLDLR) [32]. From the animal studies, Aβ clearance is highly dependent upon participant age due to the decreased abundance of LRP1 present at the BBB as the animal ages [33]. However, clinical studies have not provided significant evidence on how LRP1 may affect the development of AD.

**Figure 2:**
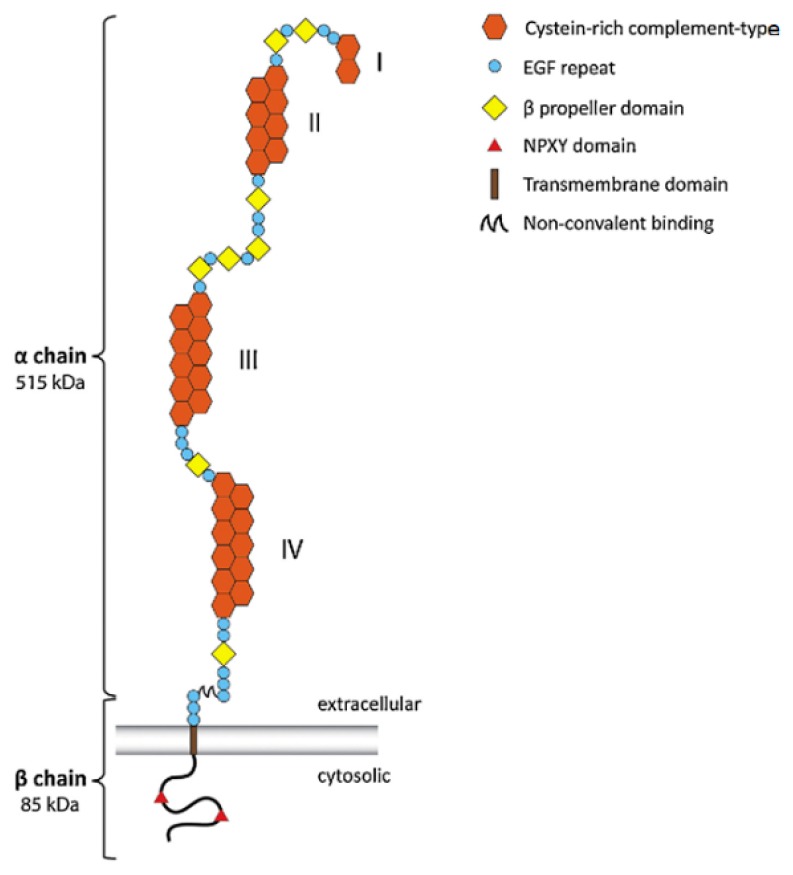
Structure of lipoprotein receptor-related protein 1 (LRP1) [38].

LRP1’s (Figure 2) distinct extracellular structures possibly identifies ligands for binding, signaling, transporting, and scavenging in the brain [12]. After LRP1 is cleaved by furin in the trans-Golgi apparatus, two subunits of LRP1 are generated and transported to the cell surface via a receptor-associated protein (RAP) [31]. These subunits of LRP1 are composed of the α and β chain noncovalently attached [33,34]. The α chain of LRP precursor is the extracellular domain containing four ligand-binding domains, I to IV, with repeats of cysteine-rich compliment types: 2, 8, 10, and 11 [32,35]. Due to its net-negative-charged domain, LRP1 attracts positively charged ligands such as Aβ, apoE, and α2M [27,36]. The calcium expedites ligands binding to both II and IV domains [31]. The β chain is the cytoplasmic domain containing the cytoplasmic tails: an NPXY motif in its cytoplasmic tail serves as a signal for endocytosis, and a YXXL motif next to NPXY expedites exocytosis [31,33]. Furthermore, receptor-associated protein (RAP) not only acts as the inhibitory ligand of LRP1, but also plays a role in the transportation of LRP1 to the cell surface, whose process is accelerated in lower pH values [31]. LRP1 and APP also share one key characteristic: they both can be cleaved by alpha- or beta-secretases, followed by gamma-secretase [31]. Through intracellular gamma-secretase cleavage, the intracellular domain of LRP1 may regulate the inflammatory gene transcription [31,37].

The mutation in the APP and PS1gene is associated mainly with the familial form of AD. Gene mutation of the LRP1 gene and/or apoE is linked to the Aβ production and clearance. In astrocytes, the genetic deletion of LRP1 has shown some features of the neurodegenerative disease related to AD, as well as other diseases, such as cancer and atherosclerosis [31,39,40]. From the knockout mouse model, the APP/PS1 with aLrp1^−/−^ mouse have shown the increase in the half-life of Aβ40 in the intracellular fluid, impairment of Aβ clearance by measuring Aβ elimination rate, and proinflammatory mediators such as interleukin-1β and TNF-α [40]. Furthermore, LRP1 controls the removal of Aβ peptides from the brain and also regulates the role of apoE gene activity. The apoE gene links to a genetic risk factor of AD, because the ε4 allele from the apoE gene has demonstrated the most potent allele risk factor [31]. Some studies suggest that apoE contributes to the Aβ accumulation in an apoE-isomer dependent manner (E4>E3>E2), while other recent clinical and preclinical studies suggest an apoE-isomer independent manner, using the LRP1-related pathway [31]. After the injection of the Aβ and apoE complex in the mouse brain, the Aβ bound to apoE2 or apoE3 is cleared by LRP1 at the BBB [31]. However, the Aβ is slowly cleared from LRP1 when it is bound to apoE4 [31]. The apoE3 produces protective effects, such as phosphorylation of a tight junction protein and occludin on the BBB, and inhibits pericyte mobility from LRP1, but those effects are deficient in apoE4 [33]. The effects of the Aβ-independent effect of apoE4 from LRP1 need further investigation.

There are several pharmaceutical and nonpharmaceutical ways to modulate LRP1 clearance. Linguizhugan decoction (LGZG), a classic traditional Chinese medicine, has been shown to decrease the Aβ accumulation in AD rats via proinflammatory cytokines, upregulation the level of LRP1, and downregulation of RAGE [41]. Statins (HMG-CoA reductase inhibitor) promote Aβ clearances from the brain by causing an increase in the level of LRP1 in the brain [42]. In clinical studies, oral administration of statins for decades demonstrated a 10%–50% reduction in the onset of AD, which could be used as a preventive method. From APP transgenic and wild-type mice, the HMG-CoA reductase inhibitor, Fluvastatin, also manifests Aβ clearance through increased levels of LRP1 [43]. The statin seems to increase the level of LRP1 in the liver, as well as in the brain. From APP/PSI mice experiment, Withania somnifera, a traditional Indian drug, has been demonstrated to increase liver LRP1, thus mediating Aβ clearance; however, further research is required [44].

## 4. CSF Circulation Assistance Device

**Figure 3:**
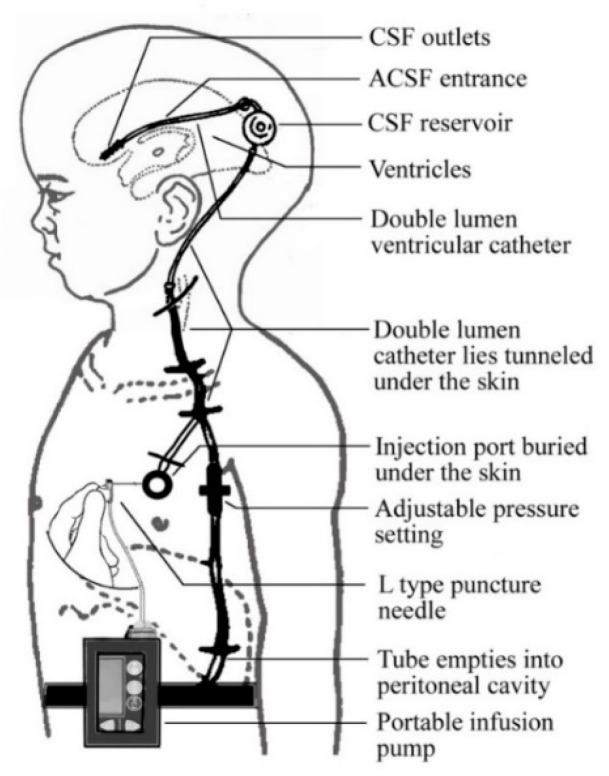
Diagram of cerebrospinal fluid (CSF) Circulation Assistant Device [45]. ACSF = artificial CSF.

Cerebrospinal fluid (CSF) circulation is required to maintain homeostasis of the internal and external environment of the brain. AD and aged patients have a decline of CSF production and an increase of outflow resistance, which can lead to interfering with CSF homeostasis and the clearance of Aβ peptides [45]. Healthy people produce ~600 mL/day of CSF, while those with AD demonstrate a reduction to ~300 mL/day [4,45,46]. With AD progression, the CSF turnover rate is reduced to 2.5 per day compared to non-AD patients of four times per day [45]. The dysfunction of CSF circulation may contribute to the disease progression of AD. A device (Figure 3) composed of an injection port, a portable infusion pump, and the ventriculoperitoneal shunt has been developed to refine CSF circulation and clearance of Aβ [45,46]. The shunt connected to the port directly goes to the ventricles of the brain [45,46]. The infusion pump connected to the injection port centrally injected can store up to 150 mL of artificial CSF (ACSF), adjust the CSF turnover rate, manage the rate of infusion, and clear neurotoxic substances [45,46]. The ACSF is designed to restore the normal CSF circulation in AD patients and thus adjust the amount of Aβ in CSF [45]. By controlling the turnover rate of CSF and ACSF, the increase in the solute concentration difference between the CSF and the brain’s interstitial fluid, is important for the efflux of soluble neurotoxic substances from the brain’s parenchyma [45]. The gradual decrease of Aβ in the CSF and ISF in the short-term may prevent the aggregation and deposits of Aβ [4]. Potentially, early stage AD patients may benefit from long-term treatment by reverting the existing deposits [4,45]. However, further clinical research and studies are required to show more benefic aspects of using a device for improving the continuous CSF circulation.

## 5. Conclusions

The rational basis of the direct clearance of Aβ to prevent the disease progression of AD is a promising therapeutic approach. The imbalance between Aβ production and removal contributes to the formation of Aβ plaques. It has been hypothesized that by increasing the clearance of Aβ from the brain, this may lead to maintaining the correct balance of Aβ. Direct targeting to receptors at the BBB facilitates the efflux of Aβ from the brain via LRP1 and/or blocks the entering of free Aβ from the peripheral circulation by antagonizing RAGE. Blocking RAGE and/or targeting LRP1 have shown benefits in animal and clinical studies by (1) the decline of proinflammatory cytokines, (2) reduction of apoptosis, and (3) improvement of cognitive abilities. Additionally, the use of a CSF Circulation Assistant Device is meant to increase the level of Aβ in the CSF by allowing the potential neurotoxic substances to diffuse out of the brain. Whether via receptors or a device, the decreased level of Aβ in the brain may prolong the amount of aggregation of the neurotoxic substances that form amyloid plaques. Thus, these new therapeutic strategies, independently, in combination, or collectively, may lead to the enhanced clearance Aβ, and thus possibly assist in delaying the disease progression of the mild-to-moderate AD patients. However, some studies had conflicting results in terms of efficacy and statistically significant, and thus further research and clinical studies are still required.

## Figures and Tables

**Table 1:** Studies evaluating Azeliragon as a RAGE antagonist.

Study Design	Result	Ref
Randomized subjects 50 years and older with mild AD with MMSE score between 12 to 26. Compare low dose PF-04494700 (10 mg), high dose PF-04494700 (20 mg), and placebo through randomization and double-blind test for 10 weeks. Measure safety by lab tests, adverse effects, vital sign, and 12-lead ECG.	There was no difference in safety issue from vital signs, laboratory results, and mean ECG.	[29]
Randomized subjects with AD and Mini-Mental State Examination score 14 to 26. Compare PF-04494700 (15 mg for 6 days, then 5 mg daily) and placebo for 18 months. Alzheimer’s Disease Assessment Scale-cognitive (ADAS-cog) used for measuring efficacy of PF-04494700.	ADAS-cog of 6 months declined greatly and discontinued due to adherence (only 50% of subjects had completed) and adverse effects. The follow up 12 months still show decline in ADAS-cog.	[28]
Compare low dose TTP488 (15 mg daily for 6 days, then 5 mg daily), high dose TTP488 (60mg daily for 6 days, then 20 mg daily), and placebo for 18 months. Efficacy measured by ADAS-cog.	By comparing the plasma concentration by using ADAS-cog, Low dose (5 mg daily) showed differences over placebo. The relationship between TTP488 plasma concentration and ADAS-cog scores was evident that low plasma concentration (7.6 to 16.8 ng/mL) was related relative decline in ADAS-cog over placebo.	[27]

AD = Alzheimer’s disease; MMSE = Mini-Mental State Examination; ECG = electrocardiography.
